# Efficacy of introducing a checklist to reduce central venous line associated bloodstream infections in the ICU caring for adult patients

**DOI:** 10.1186/s12879-018-3178-6

**Published:** 2018-06-08

**Authors:** Dominic Wichmann, Cristina E. Belmar Campos, Stephan Ehrhardt, Timo Kock, Claudia Weber, Holger Rohde, Stefan Kluge

**Affiliations:** 1 Department of Intensive Care Medicine, University Medical Center Hamburg-Eppendorf, Martinistrasse 52, 20246 Hamburg, Germany; 2 Institute for Medical Microbiology, Virology and Hygiene, University Medical Center Hamburg-Eppendorf, Martinistrasse 52, 20246 Hamburg, Germany; 3 Department of Epidemiology, Johns Hopkins Bloomberg School of Public Health, 615 North Wolfe Street, Baltimore, MD 21205 USA; 4 Schoen-Klinik Hamburg Eilbek, Dehnhaide 120, 22081 Hamburg, Germany

## Abstract

**Background:**

Central line-associated bloodstream infections (CLABSI) are a major source of sepsis in modern intensive care medicine. Some years ago bundle interventions have been introduced to reduce CLABSI. The use of checklists may be an additional tool to improve the effect of these bundles even in highly specialized institutions. In this study we investigate if the introduction of a checklist reduces the frequency of CLABSI.

**Methods:**

During the study period from October 2011 to September 2012, we investigated the effect of implementing a checklist for the placement of central venous lines (CVL). Patients were allocated either to the checklist group or to the control group, roughly in a 1:2 ratio. The frequency of CLABSI was compared between the two groups.

**Results:**

During the study period 4416 CVL were inserted; 1518 in the checklist group and 2898 in the control group. The use of the checklist during CVL placement resulted in a lower CLABSI frequency. The incidence in the checklist group was 3.8 per 1000 catheter days as compared to 5.9 per 1000 catheter days in the control group (IRR = 0.57; *p* = 0.001). The use of the checklist also reduced the frequency of catheter colonisation significantly, 36.3 per 1000 catheter days in the checklist group vs 21.2 per 1000 catheter days in the control group, respectively (IRR = 0.58; *p* < 0.001).

**Conclusion:**

The introduction of a checklist to improve the adherence to hygiene standards while placement of central venous lines reduced the frequency of infections significantly.

**Electronic supplementary material:**

The online version of this article (10.1186/s12879-018-3178-6) contains supplementary material, which is available to authorized users.

## Background

Central line-associated blood stream infections (CLABSI) are a major source of hospital acquired infections and an important contributor to the medical and financial burden in modern intensive care medicine [1–4].

Costs for a single blood stream infection are estimated at 16,500 US$ and the mortality of patients with CLABSI is 2–4 fold higher than that of similar patients without [4, 5]. Rigorous hygiene standards for placement and handling of central venous lines (CVL) are needed to prevent infections [6].

In high risk environments like aviation or nuclear power plants, checklists are standard tools to improve safety and prevent system breakdowns [7]. Knowledge transfer is not the primary intention of checklists. Pilots are trained to fly and physicians in an intensive care unit (ICU) are trained to place a CVL, thus the effect of checklists is to focus the attention of the persons involved on the actual task [7]. The use of checklists has been advocated by the World Health Organization to improve the safety of surgery and initial studies demonstrated a benefit [8]. Yet, some studies have questioned the general applicability of this approach [9]. Highly trained personnel and well established backup systems in place (e.g. blood banks, extra personnel on site, interdisciplinary support), have been put forward to explain these findings.

We aimed to assess the effect of introducing a checklist for the placement of a CVL on the frequency of CLABSI in ICU-patients in a setting of highly trained personnel in a large university hospital.

## Methods

### Setting and study design

This was an observational, prospective, single-center study at the Department of Intensive Care Medicine at the University Medical Center Hamburg–Eppendorf, Hamburg, Germany from 1st October 2011 to 30th September 2012. The department consists of 11 wards with a total of 132 ICU beds for adult patients. Each ward consists of 12 beds and is operated by a fixed team of two to three supervising senior physicians, 34 specialized nursing staff and eight assistant physicians. The department is serving for all medical and surgical specialities in need of intensive care medicine. Due to logistic reasons some wards are specialized on certain patients groups (e.g. cardiac surgery, stem cell transplantation, neurosurgery) but all teams adhere to the same standard operating procedures and there is frequent exchange of knowledge and patients between the wards. All these wards are operated on the first floor of the university campus.

To improve compliance with the Institute of Healthcare Improvement recommendations for CVL placement [10], we chose a two-step approach. In brief the measures recommended by the institute were a) hand disinfection b) full barrier nursing c) sterile disinfection of the insertion site d) avoidance of the femoral vein and e) strict indication for CVL. Physicians and nurses handling CVL placements were experienced ICU staff and intensively trained on these hygienic procedures. Training started in July 2011 with a department wide kick-off event introducing the study and theoretical backgrounds. Followed by frequent additional reminders during staff meetings on the wards to improve awareness. During the study period the decision whether or not to use the checklist for CVL placement, was made by the individual team. A ward-based team consisted of the assistant physician in charge (responsible for placing the CVL) and the assigned nursing staff (responsible for filling the checklist).

### Primary outcome

The frequency of CLABSI in both patient CVL was placed with or without the use of the checklist.

### Checklist

An English version of the checklist used in this study can be found in the (Additional file 1). In addition to the bundle components the checklist contained two formal parts for the evaluation of the study. The first part allowed to identify the setting, in detail: catheter site (jugular vs subclavian vs femoral), type (dialysis vs cvl) and urgency of placement (routine vs emergency), whereas the second part was used to document the duration of the procedure and the members of the team performing the procedure.

### Catheter types

During the study Certofix® trio-catheters (Braun, Melsungen, Germany) andMARHUKAR™ triple-lumen dialysis-catheters (Covidien™, Neustadt/Donau, Germany) were implanted.

### Patients

Data of all patients treated during the study period in one of the ICU wards with a central venous line or a temporary dialysis catheter were analyzed.

### Microbiological methods

In patients with a new episode of sepsis [11, 12] and a CVL as potential focus of sepsis, a pair of blood-cultures was taken from a peripheral site, the catheter was explanted and the tip was sent for microbiological testing. The microbiologists who had to evaluate the microbiological results had no information of the patients’ exposure status (checklist used or not used).

Blood cultures (BD Bactec PLUS Aerob/F and Bactec PLUS Anaerobe /F, Becton Dickinson, Cockeysville, USA) were incubated in a Bactec FX 40 machine for a total of five days. Material from flagged bottles was streaked onto appropriate agar media and incubated overnight at 37 °C. In parallel, aliquots taken from positive bottles were analyzed by Gram straining. All cultivated microorganisms were further differentiated to the species level by MALDI-ToF mass spectronomy (MALDI Biotyper, Bruker Daltonics, Bremen, Germany). If necessary, additional methods for species identification according to routine microbiological procedures were applied.

Explanted catheter tips were placed into 5 ml of trypticase soy broth and incubated at 37 °C without shaking. Cultures were visually inspected for growth on a daily basis. Any cultures suspected for microbial growth was streaked onto agar media (Columbia agar containing sheep blood [5% *v*/v]; McConkey agar; Sabouraud agar, all Oxoid, Basingstoke, UK). Microorganisms were further differentiated as outlined above.

The broad use of antimicrobial substances in an ICU setting reduces the sensitivity of blood cultures by about 36% [12–14]. Therefore statistical analyses were performed stratified by CLABSI and colonized CVL.

#### Definition of CLABSI

Corresponding positive microbiological cultures from the catheter tip and a blood culture taken from a peripheral site at the time of the device explantation in a patient with a new onset of sepsis and a CVL as potential sepsis focus [15].

#### Definition of colonized CVL

A positive microbiological culture from the catheter tip but no corresponding blood culture taken from a peripheral site at the time of the device explantation in a patient with a new onset of sepsis and a CVL as potential sepsis focus [15].

#### Definition of sepsis

Sepsis was defined according to the international guidelines of the Surviving Sepsis Campaign [16].

### Statistical methods

We first computed descriptive statistics like counts, frequencies, and incidences per 1000 catheter days. Incidence rate ratios (IRRs), 95% confidence intervals (CIs) and *P*-values were calculated using STATA 12.1.

## Results

During the study period 4416 CVL were implanted, 1518 in the checklist group and 2898 in the control group. Patient characteristics in both groups did not differ significantly with respect to age (64.7 ± 14.4 checklist group; 64.8 ± 14.8 control group), male to female ratio (2:1), patient type (surgical, medical), disease severity, or length of ICU stay.

We identified CLABSI in 39 of 1518 patients contributing 11,540 catheter days (3.8 per 1000 catheter days) in the checklist group and in 127 of 2898 patients contributing 21,349 catheter days (5.9 per 1000 catheter days) in the control group (IRR 0.57, 95% CI 0.39–0.82, *P* = 0.001).

When analyzing colonized CVL, we detected 245 events in 1518 patients (11,540 catheter days; 21.2 per 1000 catheter days) in the checklist group and 776 events in 2898 patients (21,349 catheter days; 36.3 per 1000 catheter days) in the control group (IRR 0.58, 95% CI 0.50–0.68, *P* < 0.001).

When analyzing the formal aspects of the checklist we identified 267 checklists (17.6%), where at least one of the paragraphs was filled out incompletely, suggesting incomplete compliance. An example for a checklist obviously filled out after the procedure, is available in the (Additional file 2). Nevertheless patients with incomplete checklists had the same reduction in CLABSI as patients with complete checklists. These effects were consistent over all types of ICUs (surgical, medical, neurological); data not shown. In two ICUs patients had a much below average length of stay (< 3 days), with very few newly placed CVLs. Thus here a statistical evaluation of the adherence to the checklist was not possible.

**Fig. 1:**
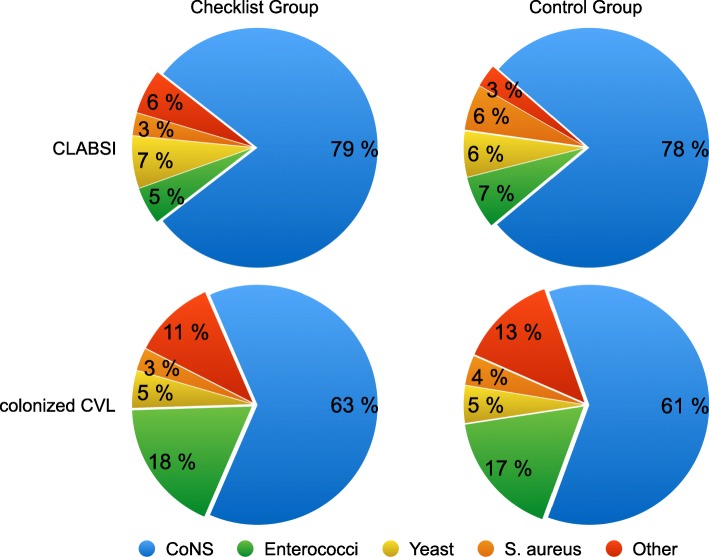
Distribution of isolated pathogens in the checklist group and the control group for CLABSI and colonized CVL

For CLABSI the majority of pathogens isolates were Coagulase negative *Staphylococci* (CoNS) (79%), *Enterococci* (5%), *S. aureus* (3%) and yeasts (7%) in the checklist group and CoNS (76%), *Enterococci* (7%), *S. aureus* (6%) and yeast (6%) in the control group. For colonized CVL the distribution was CoNS (63%), *Enterococci* (18%), *S. aureus* (3%) and yeasts (5%) in the checklist group and CoNS (61%), *Enterococci* (17%), *S. aureus* (4%) and yeasts (5%) in the control group (Fig. 1**)**. When analyzing the CLABSI no significant differences were detected for the site of catheter insertion. Data for the site of insertion were available for *n* = 1249 catheters; jugular vein (*n* = 710), subclavian vein (*n* = 272), femoral vein (*n* = 267). Catheters placed in the jugular vein had an infection rate of 3.6 per 1000 catheter days compared to 2.7 per 1000 catheter days for all other sites (IRR 1.33, 95% CI 0.60–3.02, P 0.23). Subclavian vein catheters had an infection rate of 1.3 per 1000 catheter days compared to 3.4 per 1000 catheter days for all other sites (IRR 1.04, 95% CI 0.40–2.43, *P* = 0.45). Femoral vein catheters had an infection rate of 2.0 per 1000 catheter days compared to 7.8 per 1000 catheter days for all other sites (IRR 0.58, 95% CI 0.15–1.67, *P* = 0.16).

**Fig. 2:**
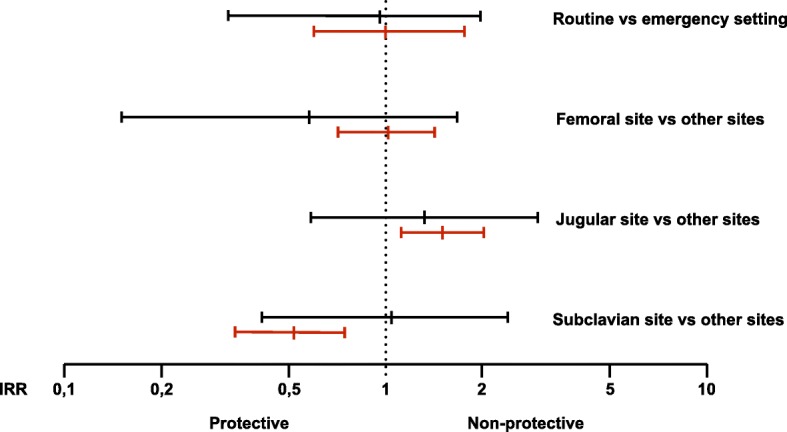
Incidence rate ratios (central vertical line) and 95% confidence intervals (horizontal lines) for various catheter placement sites compared to their comparators. Values for CLABSI are displayed in black, values for colonized CVL in red. Values smaller “1” demonstrate a protective effect, larger “1” a non-protective. Incidence rate ratios for emergency vs routine setting are shown accordingly

For colonized CVL a protective effect for catheters placed in the subclavian vein was seen (infection rate 5.1 per 1000 catheter days vs 23.5 per 1000 catheter days compared to other sites; IRR 0.52, 95% CI 0.34–0.76, *P* = 0.0001). When analyzing potential effects of the setting in which the catheter was placed (emergency vs routine) no significant differences were detected for both CLABSI definitions (Fig. 2**)**.

## Discussion

Introduction of a checklist to improve compliance with hygiene standards for CVL placement in a high volume intensive care setting lead to a significant reduction in CLABSI. Benchmarking with other German hospitals in 2010/11 demonstrated that we ranked in the upper half for CLABSI-incidence in the ICU before introducing the checklist. CLABSI contribute substantially to healthcare cost [2]. The reduction of hospital acquired infections has been found cost effective [17, 18]. According to conservative estimates, the cost of a single CLABSI-episode is about 16,500 US$ [5]. Use of the checklist would thus have resulted in savings of more than 0.9 Mio US$ per year in our study population. Although our study was not designed to demonstrate an effect on mortality it seems a reasonable assumption that the reduction in CLABSI frequency of about 40% would have resulted in a notable improvement of patient safety and outcomes [4, 5]. Thus our study demonstrates that checklists may improve the quality of standard tasks associated with a relatively high proportion of complications. All team members were constantly educated on advantages of checklists as well as the special content and intention of our checklist and the aim of our study in particular. Interestingly, completing the checklist for a standard ICU task had a significant effect on the reduction of CLABSI, even if the checklist was used with incomplete compliance. For example a checklist obviously filled out after the procedure had the same effect on reducing the CLABSI rates as a checklist filled out correctly. One might speculate that the team is focusing on the task if they are aware of the existence of the checklist even if they do not actually use it during the task itself. This may explain why checklists have generally been shown to improve the performance of even highly trained personnel when performing tasks under stress [7]. The Institute of Healthcare Improvement has promoted recommendations for CVL placement to prevent CLABSI [10]. Checklists have been used in various settings (pediatric/surgical ICUs, developed) with generally positive results [19–21]. Yet, the use of checklists in highly developed and high volume health care settings has been questioned by recent data from Urbach and colleagues [9]. Failure to demonstrate an advantage of checklists may, however, be due to the endpoint mortality. Differences in mortality may be difficult to demonstrate in a well developed health care system, when rescue measures are in place to deal with potentially hazardous situations like bleeding, shock, or infections. For the prevention of CLABSI the results of our study are in line with findings of other groups [19, 22]. Pronovost and colleagues used a multicenter approach together with the state health system of Michigan. Each participating ICU chose a nurse and a physician as multipliers to distribute the information to the ICU staff, local infection control teams gave monthly feedback about infection rates and measures to improve a safety culture were implemented by periodic team meetings and conferences. The adherence to the infection-control practices was supported by the use of checklist [22]. A similar project reported by DePalo and colleagues from ICUs in Rhode Island highlights the need for the implementation of a local safety culture and the designation of local team leaders and the commitment for ongoing improvement by quality improvement cycles (Engagement, Education, Execute and Evaluate) [19].

In clinical practice the decision whether or not to treat inconclusive microbiological results (colonized CVL) is often difficult, particularly if common skin flora like CoNS, *S. aureus* or yeasts are isolated [23, 24]. Contamination of the catheter tip during explantation might be one explanation, inoculation during catheter placement with concomitant colonization and infection the other. Most of our patients were critical ill patients and received antibiotic treatment for various reasons (secondary/tertiary peritonitis after abdominal surgery, hematological/oncological patients with sepsis, patients with neurological diseases and hospital acquired pneumonia). Pazin and colleagues showed that the sensitivity of blood cultures drops significantly in a setting like this [13].

In our study the catheter site (jugular/subclavian/femoral) had no effect on the frequency of CALBSI episodes. This is in contrast to older studies and current guidelines [6]. Meta-analyses however [25], suggested that site-specific effects may be due to two studies with extreme results [26, 27]. When excluding these studies from the analysis no effect of the insertion site with regard to infection frequency was observed [25, 28].

On the other hand we could demonstrate a protective effect for the subclavain vein insertion site compared to all other sites in the stratum of colonized CVL, which has also been shown by Parienti [29]. This is most likely explained by the fact that the subclavian route has the longest subcutaneous distance between skin and vessel entry. However regarding this issue our results are difficult to interpret because of unstable estimates due to the relatively low numbers of catheters included in this analysis and a large number of catheters where the site of placement was not documented.

Our study has limitations. First, the lack of randomization in this observational study may have introduced confounding. On the other hand all participants were instructed on the content of the checklist even if the CVL was implanted without using it. This may have resulted in an underestimation of the effect of the intervention. Second, this was a single center experience of a high-volume ICU without specialized “CVL teams”. Thus, while we believe our setting is not unusual, caution is needed when generalizing our findings to other settings.

## Conclusion

Our data suggest that a checklist is a valuable tool to prevent CLABSI in ICU patients, may improve patient safety, and subsequently reduce costs for hospital acquired infections. The implementation of checklists for CVL placement should be encouraged even when performed by highly trained ICU personnel.

## Additional files

Additional file 1: **Figure S1.** The English version of the checklist used for this study. (DOCX 191 kb)

Additional file 2: **Figure S2.** An example of a checklist, obviously filled out after the procedure. All checks are made with a single dash. The time for the procedure, including preparation, is documented with only 10 min. (PDF 4552 kb)

## Abbreviations

CLABSI: Central line-associated blood stream infection
CoNS: coagulase negative staphylococci
CVL: Central venous line
ICU: Intensive care unit
IRR: Incidence rate ratio
MALDI-TOF: Matrix assisted laser desorption ionization - time of flight
