# Supplementary material for: Efficacy of introducing a checklist to reduce central venous line associated bloodstream infections in the ICU caring for adult patients
Source: BMC Infect Dis. 2018 Jun 8;18:267. doi: 10.1186/s12879-018-3178-6 (PMC5994052; doi:10.1186/s12879-018-3178-6)
Supplement: Supplementary file 1 — Figure S1. The English version of the checklist used for this study. (DOCX 191 kb) [file 12879_2018_3178_MOESM1_ESM.docx]

**
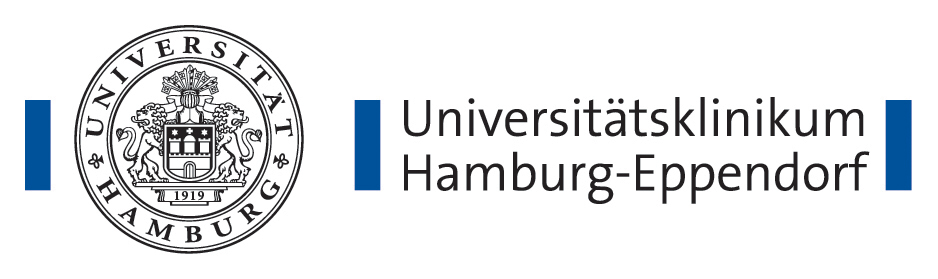
**

**Central venous catheter checklist**

**Setting:**

routine ◯ emergency ◯

new ◯ reposition ◯

cvl ◯ dialysis ◯

jugular ◯ subclavian ◯ femoral ◯

right ◯ left ◯

Done Done after

independendly reminded

**Preparation:**

Putting on surgical hat ◯ ◯

Putting on face mask ◯ ◯

Assisting person putting on face mask ◯ ◯

Scrub disinfection of insertion site ◯ ◯

**Clothing/Hand hygiene:**

Putting of watch/ring/braclet/etc. ◯ ◯

Washing hands with soup ◯ ◯

Hands dried ◯ ◯

Hand disinfection with alcohol based disinfectant (3min) ◯ ◯

Putting on steril coat ◯ ◯

Coat closed on the back ◯ ◯

Putting on steril gloves ◯ ◯

Returning the coats checkcard ◯ ◯

**Preparation of the patient:**

Patients skin dried ◯ ◯

Full steril draping in place (including borders of the bed) ◯ ◯

**Meta-data:**

Date:

Starting time: Finishing time:

Physician: Assisting person:

Signature _---------------------------------------------_ Signature _---------------------------------------------_
