# Supplementary material for: Efficacy of introducing a checklist to reduce central venous line associated bloodstream infections in the ICU caring for adult patients
Source: BMC Infect Dis. 2018 Jun 8;18:267. doi: 10.1186/s12879-018-3178-6 (PMC5994052; doi:10.1186/s12879-018-3178-6)
Supplement: Supplementary file 2 — Figure S2. An example of a checklist, obviously filled out after the procedure. All checks are made with a single dash. The time for the procedure, including preparation, is documented with only 10 min. (PDF 4552 kb) [file 12879_2018_3178_MOESM2_ESM.pdf]

Universitätsklinikum
Hamburg-Eppendorf

Central venous catheter checklist

Setting:

routine	O	emergency	O		
new	O	reposition	O		
cvl	O	dialysis	O		
jugular	O	subclavian	O	femoral	O
right	O	left	O		

	Done independendly	Done after reminded
**Preparation:**		
Putting on surgical hat	O	O
Putting on face mask	O	O
Assisting person putting on face mask	O	O
Scrub disinfection of insertion site	O	O
**Clothing/Hand hygiene:**		
Putting of watch/ring/braclet/etc.	O	O
Washing hands with soup	O	O
Hands dried	O	O
Hand disinfection with alcohol based disinfectant (3min)	O	O
Putting on steril coat	O	O
Coat closed on the back	O	O
Putting on steril gloves	O	O
Returning the coats checkcard	O	O
**Preparation of the patient:**		
Patients skin dried	O	O
Full steril draping in place (including borders of the bed)	O	O

Meta-data:

Date: 05/03/12

Starting time: 03 40

Finishing time: 03 50

Physician: [redacted]

Assisting person: [redacted]

Signature [redacted]

Signature [redacted]
